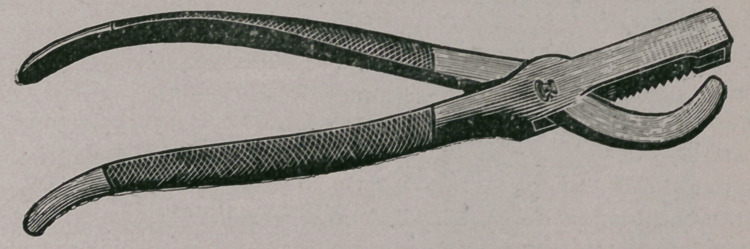# Emasculator

**Published:** 1891-02

**Authors:** 


### HAUSSMANN EMASCULATOR.

Messrs Haussmann, McComb & Dunn of Chicago have recently produced a castrating forceps which allows of rapid use, shown by the accompanying illustration.

The lower blade is solid and passes through the upper blade which is fenestrated. One edge of the upper blade is rough and the other edge sharp; the roughened edge is slightly raised so that in closing the blades, the cord is engaged between the lower blade and the roughened edge of the upper blade, and crushed before reaching the cutting edge.

## Figures and Tables

**Figure f1:**